# P-897. Impact of a multi-faceted stewardship intervention on selection of guideline-concordant antibiotics for surgical prophylaxis in a large hospital system

**DOI:** 10.1093/ofid/ofaf695.1105

**Published:** 2026-01-11

**Authors:** Natalie Finch, Wesley J Hoffmann, Shivani Patel, Shemual Tsai, Muhammad Yasser Alsafadi

**Affiliations:** Houston Methodist Hospital, Houston, TX; Houston Methodist Hospital, Houston, TX; Houston Methodist, Houston, Texas; Houston Methodist Hospital, Houston, TX; Houston Methodist, Houston, Texas

## Abstract

**Background:**

Guideline adherence for antibiotic surgical prophylaxis is low nationwide, with use of non-preferred agents linked to worse outcomes. Patients with reported penicillin allergies are less likely to receive cefazolin - the guideline-preferred antibiotic for most surgical procedures- and more likely to receive non-preferred antibiotics. To address this, we implemented a multi-faceted antimicrobial stewardship intervention across a large, multi-hospital healthcare system and evaluated the impact of three distinct interventions on surgical prophylaxis antibiotic selection.

**Figure 1:**
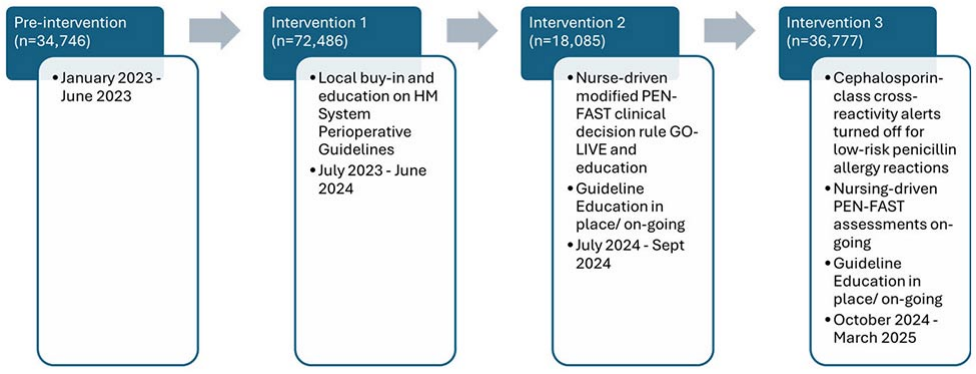
Interventions and Time Periods

**Figure:**
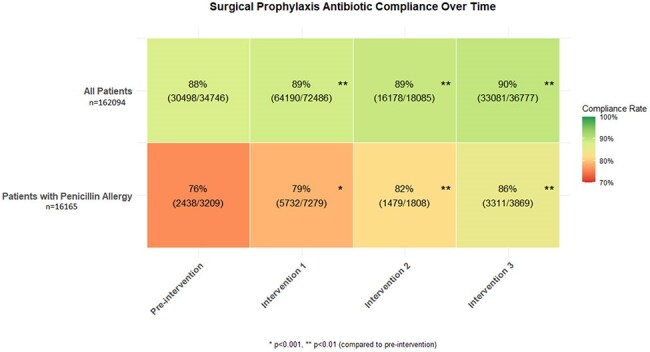
Surgical Prophylaxis Antibiotic Compliance Over Time

**Methods:**

We conducted a retrospective cohort study of adults (≥18 years) who underwent a surgical procedure requiring antibiotic prophylaxis across the eight hospital Houston Methodist (HM) Healthcare System (July 2023-March 2025). Only the first procedure per encounter was included. Patients were grouped by intervention period and evaluated for adherence to recommended antibiotic regimens within the HM System Guidelines for Surgical Prophylaxis (Figure 1).The primary outcome was overall guideline regimen adherence. Secondary outcomes included guideline regimen adherence and incidence of cefazolin use among penicillin allergic patients and incidence of non-beta-lactam use in patients with low-risk PEN-FAST scores (score 0-2).

**Figure 3:**
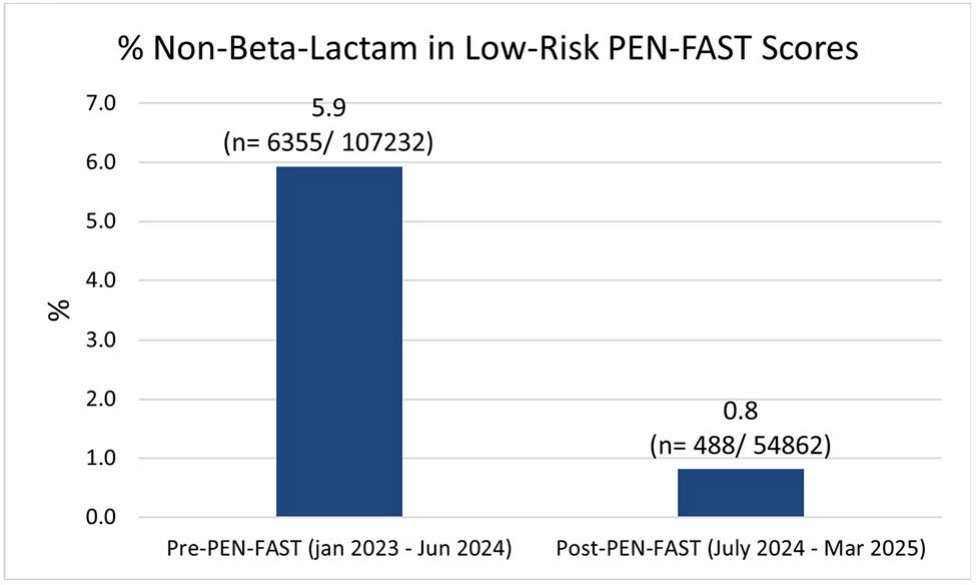
Non-Beta-Lactam Use in Low-Risk PEN-FAST Scores

**Results:**

Surgical prophylaxis antibiotic compliance improved significantly after each intervention compared to the pre-intervention period in both the overall and penicillin-allergic populations (Figure 2). In penicillin-allergic patients, cefazolin use increased from 56.8% to 71.0% (p< 0.001), demonstrating an increase in first-line regimen selection despite presence of a penicillin allergy. After implementing a nurse-driven PEN-FAST screening and adjusting cephalosporin cross-reactivity alerts, non-beta-lactam use in low-risk penicillin allergy patients (PEN-FAST score 0-2) significantly declined (p< 0.001; Figure 3).

**Conclusion:**

A multifaceted antimicrobial stewardship intervention including frontline clinician education, penicillin allergy assessment, and penicillin- cephalosporin cross-reactivity alert modifications effectively increased preferred antibiotics for surgical prophylaxis.

**Disclosures:**

All Authors: No reported disclosures

